# Experimental evidence that the perennial grass persistence pathway is linked to plant growth strategy

**DOI:** 10.1371/journal.pone.0207360

**Published:** 2018-11-26

**Authors:** Paulo Gonçalves Duchini, Gabriela Cristina Guzatti, Joilson Roda Echeverria, Luana Fidelis Américo, André Fischer Sbrissia

**Affiliations:** Animal Production and Food Science Department of Santa Catarina State University (UDESC/CAV), Avenida Luiz de Camões, Lages, SC, Brazil; Helmholtz Centre for Environmental Research - UFZ, GERMANY

## Abstract

Grass species can be classified into different functional types based on their growth strategies, and contrasting persistence strategies are observed in different grass species. Excluding seedling recruitments, changes in populations of grasses are basically a trade-off between natality and mortality of tillers. We hypothesised that the persistence pathway of perennial grasses is linked to their growth strategy, regardless whether they are growing as monoculture or as a mixture. Species with contrasting growth strategies (*Arrhenatherum elatius* L., *Dactylis glomerata* L., and *Festuca arundinacea* Schreb.) were cultivated as monocultures and as a mixture and their tiller natality and mortality were evaluated for two years after swards establishment. All pastures maintained their population size during the experimental period, although decreases in tiller densities occurred during the warmer season. *Arrhenatherum elatius* had the highest tiller mortality and natality whereas the *F*. *arundinacea* had the lowest ones. *Arrhenatherum elatius* had many tillers appearing in all seasons but their tillers were short-lived. Conversely, *F*. *arundinacea* and *D*. *glomerata* developed numerous tillers during autumn and winter and their tillers survived, on average, almost six and three times longer than those of *A*. *elatius*, respectively. There were no differences in tillering dynamics among populations grown in monocultures or in the mixture. Regardless of whether they were cultivated in monocultures or as a mixture, the persistence pathway of perennial grasses is linked with their growth strategies with exploitative species presenting a high tiller turnover throughout the year whereas the persistence of more conservative species is based on a high tiller survival.

## Introduction

Different schemes aimed to group plant species according to their functioning and ecological strategies, such as *r-K* continuum [1], C-S-R [2], and leaf-height-seed theories [3]. These different groups of plants present a common pool of traits that defines their strategies based on at least three fundamental axes: the resources use and acquisition, the competitive ability and the plant capacity for sexual reproduction [3, 4]. For [3], specific leaf area, plant height, and seed mass can be used to describe these axes, which are commonly used to assess the plant strategies in natural ecosystems (e.g. rangelands). However, in agronomic cultivated perennial grasslands a very high sward height and flowering are relatively undesirable and can be controlled by grazing managements practices [5]. Moreover, seedling recruitment is rare in perennial grasses [6] and their vegetative reproduction play a critical role in aboveground net primary production [7] and in pasture resilience and persistence over a range of environments [8]. In this scenario, the species ability to capture and use the available resources and its capacity for vegetative reproduction (tillering from the bud bank [9]) could be seen as the main features to be considered when evaluating species in cultivated grasslands.

According to [10], grassland species, including different C3 grasses [11], can be classified into different ‘functional types’ depending on their growth strategies. In this sense, the most exploitative species have a greater ability to use available resources and renew their tissues than more conservative ones do and, therefore, they have a greater amount of buds capable of generating new tillers [12]. A fast-slow continuum was already showed as the main axes determining life-history in plants from natural populations [13]. However, tiller mortality and natality are under hormonal, genetic, environmental, and management controls [14, 15]. Therefore, the lower the resource limitation (stress) and disturbance, the greater the influence of intrinsic factors on vegetative reproduction (tillering dynamics), in such a way that how species maintain its population stable can be strongly influenced by their growth strategies.

[16] compared tillering dynamics of ten forage grass species and highlighted dissimilarities in their persistence strategies. Some of these species expressed seasonal tillering influenced by flowering and/or weather conditions, whereas others had continuous tillering throughout the year. Thus, population stability and pasture persistence can occur by high tiller natality, high tiller survival or both over time [17]. In this way, indexes as the per capita rate of population change (*r*) or population stability index (P_1_/P_0_) allow an integrative analysis of these two variables and makes it possible to understand the importance of tiller natality and tiller survival in the persistence of plant populations [18, 19].

[16] highlighted that there is a lack of information regarding tillering dynamics in agronomical multispecific swards and that such studies would be important to a better understanding of plant population ecology. This is reinforced by the recent recommendation of utilising multispecies pastures not only to increase the ecosystem multi-functionality [20] but also to enhance the forage production and stability in intensive pasture-based animal production systems [21]. Mixing three species that are well-adapted to the environmental and management conditions appears sufficient in the formulation of productive agronomic mixtures to reach the benefits promoted by biodiversity [22, 23]. Therefore, choosing species with contrasting growth strategies (as discussed above) could be a valuable option to formulate mixtures to be used in animal production systems. That is supported by works that show to be possible the coexistence of these species in fertile environments [24], especially when light competition is minimised by defoliation [25]. However, to promote the persistence of these species, it is a requirement that the tillering ability and maintenance of a stable population over time are not harmed when species are mixed, which could be allowed by using managements strategies that minimize stresses and disturbances that knowingly would displace exploitative (competitor) and conservative species (stress-tolerant), respectively [2, 26].

We hypothesised that exploitative (i.e fast growing) and conservative (i.e slow growing) perennial grasses managed to minimize stress and disturbance have their persistence pathway linked with their growth strategy, regardless whether they are growing as monoculture or as a mixture. Three model species differing in growth strategy were chosen to test our hypotheses: an exploitative species (*Arrhenatherum elatius* L.), a conservative species (*Festuca arundinacea* Schreb.), and a moderately exploitative species (*Dactylis glomerata* L.) based on information reported by [27, 28]. These three species were sowed in monocultures and as a mixture and were managed during one year for the pastures establishment and another two years for data collection.

## Material and methods

### Experimental area

The experiment was conducted at the Centre of Agriculture and Veterinary Sciences, of the Santa Catarina State University, in Lages, Santa Catarina, Brazil (27°47′ S, 50°18′ W, 960 m above sea level). The climate in the region of the experiment is humid subtropical under oceanic influences (Cfb) based on the Köppen classification system, with cool winters and mild summers, and rainfall well-distributed throughout the year [29]. The average annual rainfall is 1,543 mm and the average temperature varies between 11°C in July and 20.4°C in January. During the experimental period temperature was close to and rainfall was never lower than historical values (S1 Fig). The experimental area was prepared in April 2013 in a inceptisol soil (Cambissolo Húmico Alumínico Típico) that is native to the region, with the following characteristics at a depth of 0–20 cm: pH (Shoemaker-McLean-Pratt [SMP]) = 4.3, organic matter = 2.1%, K = 48 mg dm^-3^, P = 3.6 mg dm^-3^, Ca = 1.16 cmol_c_ dm^-3^, Mg = 0.82 cmol_c_ dm^-3^, H + Al = 30.7 cmol_c_ dm^-3^, cation exchange capacity at pH 7.0 = 32.8 cmol_c_ dm^-3^, base saturation = 6.4%, and clay = 52.0%. In 4 June 2013, liming and fertilisation applications were performed with dolomitic limestone, single superphosphate, and potassium chloride (at half the recommended amount) based on procedures described in the Manual of Fertilisation and Liming for the Rio Grande do Sul and Santa Catarina States to maintain a highly fertile environment [30].

### Treatments and management

The experimental area was divided into 12 plots of 45 m^2^ (experimental units). The corridors between the plots and the surrounding experimental area contained no vegetation. Four treatments were repeated three times and were randomly distributed within the plots on 13 June 2013. The treatments were *A*. *elatius* ‘SCS314 Santa Vitória’, *F*. *arundinacea* ‘Quantum II’, and *D*. *glomerata* ‘Ambar’ sowed as monocultures and as a mixture composed of the three species in the same proportions. These three species were chosen because of their distinct growth strategies (competitive abilities). *Arrhenatherum elatius* is an exploitative species (with high specific leaf area, leaf nitrogen content, and tissue renewal), *F*. *arundinacea* is a conservative species (with low leaf area, leaf nitrogen content, and tissue renewal), and *D*. *glomerata* is a moderately exploitative species with intermediary characteristics compared to the other species [24, 27, 28]. Moreover, despite of their contrasting growth strategies, these species are potentially dominant and can produce high yields in monoculture or mixtures [23, 31, 11].

Sowing was carried out by broadcasting on 14 June 2013 at a seeding rate of 18 kg ha^-1^ of pure viable seeds (commercial recommendation of 15 kg ha^-1^ + 20% for broadcast sowing), and the species ratio was 1/3 for each species in the mixture treatment. Following sowing pastures were maintained under free growth for the establishment until March 2014. During this period a second application of potassium and the first application of nitrogen fertilisation with 70 kg of N ha^-1^ as urea were applied. In March 2014, a motor scythe was used to cut the pastures to 7 cm above the soil surface to stimulate pasture renewal and the second application of nitrogen (50 kg of N ha^-1^ as urea) was applied after the cutting. Two months later another cut was made at a height of 10 cm to commence the data collection period that ran from May 2014 to August 2016.

In May 2014, when the pastures reached 20 cm in height (considered the pre-cut height), they were lowered to 10 cm by a brush cutter (considered the post-cut height, based on a defoliation intensity of 50%) and all cut material was removed from the plots. The pre-cut height was chosen to correspond to the canopy condition that intercepted 95% of the incident radiation (measured with a AccuPAR LP-80 ceptometer; Decagon Devices Inc., Pullman, WA, USA) during the full vegetative developmental stage (from March to September 2014) for the three studied species. Sporadic evaluation of light interception was carried out when plots reached 20 cm in height to ensure that the pastures did not intercept more than 95% of the incident radiation during the experimental period. The pre- and post-cut heights were monitored by measuring 20 points per plot using an acetate sheet and ruler before and after cutting.

Soil analyses were performed separately for each treatment in late autumn and fertilisers were added when necessary to maintain a highly fertile environment [30]. The phosphorus and potassium quantities were divided and applied in 2–4 fertilisations per year, whereas nitrogen fertilisations were carried out with urea every 30–60 days (depending on the rainfall) throughout the experimental period with 30 kg of N ha^-1^ (in 2014/2015) and 50 kg of N ha^-1^ (in 2015/2016) to maintain nitrogen nutrition indices close to the N_critic_ [32]. Fertilisation activities are described in greater detail in the supporting information (S1 Table). The amount of nitrogen applied in 2014/2015 was determined based on previous studies [32]. However, it was decided to increase the nitrogen doses in 2015/2016 because the nitrogen nutrition index was close to the lower threshold for the three species (unpublished data).

Fungicides were used to control leaf spot and rust, and insecticide applied to control *Collaria scenica* (Hemiptera: Miridae) when necessary. Plants that were not the studied species were removed by manual pulling.

### Measurements

Tiller density was measured in two 70 × 20 cm frames located at sites representative of the average canopy condition at the cutting time. The material inside the frames was cut at ground level and taken to the laboratory to count the tiller density. In the mixture a botanical separation by species was performed, and then the number of tillers of each species was counted. Most of the dead material had detached from the tillers and it was impossible to identify it to species level. Therefore, all the dead material including that was still attached to the tillers was quantified together. Then, all tillers (without dead material) were oven-dried with forced air circulation at 65°C for 72 hours to determine tiller weight.

The tiller population dynamics were evaluated in two 20-cm-diameter circles (0.0314 m^2^) per plot that were fixed randomly during the last week of May 2014. Twenty-two evaluations were undertaken from June 2014 to July 2016. During the first evaluation all tillers contained inside the rings were marked with distinct colourful plastic clips for each species (this tillers were considered as belonging to cohorts *a*). In subsequent evaluations new colour clips were used to identify the newly emerged tillers. Different colours were used to identify which species (in the mixture) and cohort each tiller belonged to. Simultaneously the dead tillers of each species and generation were recorded and their clips were removed. Withered and brown/yellow tillers were considered to be dead.

Tiller natality, tiller mortality, and tiller survival of *A*. *elatius*, *F*. *arundinacea*, and *D*. *glomerata* populations in monoculture and in the mixture were calculated. Tiller natality (tillers 100 tillers^-1^) was considered as the tiller number that appeared between two evaluations in relation to the total tiller population in the previous evaluation. Tiller mortality (tillers 100 tillers^-1^) was similarly calculated but the number of tillers that had died during the same period was used. Thus, tiller survival (tillers 100 tillers^-1^) was determined by subtracting the tiller mortality from 1. Tiller natality, tiller mortality, and tiller survival were also calculated for the mixture by counting the tiller number that appeared and died from each of the three species. The values were adjusted for a 30-day period.

The per capita rate of population change (*r*) was calculated according to [18]:

r=b−d

where, *b* and *d* are the per capita tiller natality and mortality, respectively. This metric *r* provides an overview of the stability of tiller populations between successive evaluations. Tiller population increases when *r* > 0, and decreases if *r* < 0. Based on this metric if a pasture has, for example, a *r* equal to 0.2, this means that there was a 20% increase in tillers during that period.

Moreover, based on [19], the population stability indexes were also calculated as follow:

$$P_{1}/P_{0}=\left(b+s\right)t_{1}‑t_{0}$$

where, P_1_/P_0_ is the proportion of the population of tiller existing in the current evaluation (t_1_) and previous one (t_0_); *b* and *s* are per capita tiller natality and survival, respectively, during the evaluated period. Similar as in per capita rate of population change, the population stability index also indicate changes in population size, but P_1_/P_0_ > 1 show increases and P_1_/P_0_ < 1 decreases in tiller population.

Diagrams of the tillering demography were also generated to indicate the number of tillers that appeared in each cohort and its decrease along the successive evaluations. The half-life of tillers from each cohort was calculated for monocultures based on the equation described by [33]:

$$t_{1/2}=ln\;2/b$$

where, *t*_*1/2*_ is the half-life of a tiller cohort and *b* is the regression coefficient for exponential regression resulting from a reduction in the number of tillers of this generation over time. Subsequently, the average half-life of tiller cohorts appearing in each season was calculated and weighted using the number of tillers from each of these cohorts.

### Statistical analysis

The observations from the two circles or the two frames of each one of the plots were been pooled together. Because the cuttings applied to the different treatments were based on height, the times of the cuttings were not the same for all treatments during the experimental period. Since the harvests were performed in a non-chronological time, the measured variables were therefore calculated by season of the year to allow for comparisons between the studied variables under the same conditions. Simple linear interpolations were used to estimate seasonal tiller mortality, tiller natality, tiller survival, and *r* values. The seasonal values for the other variables were the average of all cuts performed during each season. The seasonal data were examined by analysis of variance using the MIXED procedure (mixed models) of SAS, version 9.2 (SAS Institute, Cary, NC, USA). Akaike information criterion was used to choose the covariance matrix that best fitted to the datasets. The models included the treatment (T), year (Y), season (s), and the treatment × year (T*Y) and treatment × season (T*s) interactions as described below:

$$y_{ijk}=\mu +T_{i}+Y_{j}+s_{k}+\left(T*Y\right)+{\left(T*s\right)}_{ik}+\varepsilon_{ijk}$$

The means were estimated using the least-squares means method and differences between them were compared by Tukey’s test at *P* < 0.05.

## Results

### Tiller density and tiller weight

The highest tiller densities were observed in *F*. *arundinacea* and *A*. *elatius* (Table 1). *Dactylis glomerata* and the mixture had the lowest tiller densities during all seasons. There was no year effect on tiller density (*P* = 0.88); however, the highest tiller densities were observed during the autumn and winter in *A*. *elatius*, *D*. *glomerata*, and the mixture, whereas *F*. *arundinacea* had its highest tiller densities during winter and spring (*P* < 0.01). Conversely, heavier tillers were usually associated with lower tiller densities in all treatments (*P* < 0.01; Table 1).

**Table 1:** Tiller density and tiller weight of *Arrhenatherum elatius*, *Festuca arundinacea*, and *Dactylis glomerata* swards sowed as monoculture or as a mixture throughout the seasons.

Treatments	Winter	Spring	Summer	Autumn	Mean
	*Tiller density (tillers m*^*-2*^*)*				
*Arrhenatherum elatius*	3096 ab A	2027 b B	2420 a B	2510 a AB	2513
*Festuca arundinacea*	3571 a A	3291 a A	2357 a B	2291 a B	2878
*Dactylis glomerata*	2404 c A	1779 b B	1715 b B	1919 a AB	1954
Mixture	2718 bc A	2136 b B	1763 b B	2302 a AB	2230
Mean	2947	2308	2064	2256	
	*Tiller weight (mg DM tiller*^*-1*^*)*				
*Arrhenatherum elatius*	66.5 b B	92.1 a A	76.4 b AB	63.1 b B	74.5
*Festuca arundinacea*	82.6 ab B	82.1 a B	111.6 a A	92.0 a AB	92.1
*Dactylis glomerata*	97.0 a A	106.6 a A	107.4 a A	93.3 a A	103.3
Mixture	86.3 ab B	101.5 a AB	123.6 a A	101.7 a AB	101.1
Mean	83.1	95.6	104.7	87.5	

Means followed by the same uppercase letter in rows and lower case in columns are not significantly different (P > 0.05).

SEM of treatment × season interaction = 116.5 and 5.03 for tiller density and tiller weight, respectively.

### Tillering demography

**Fig 1:**
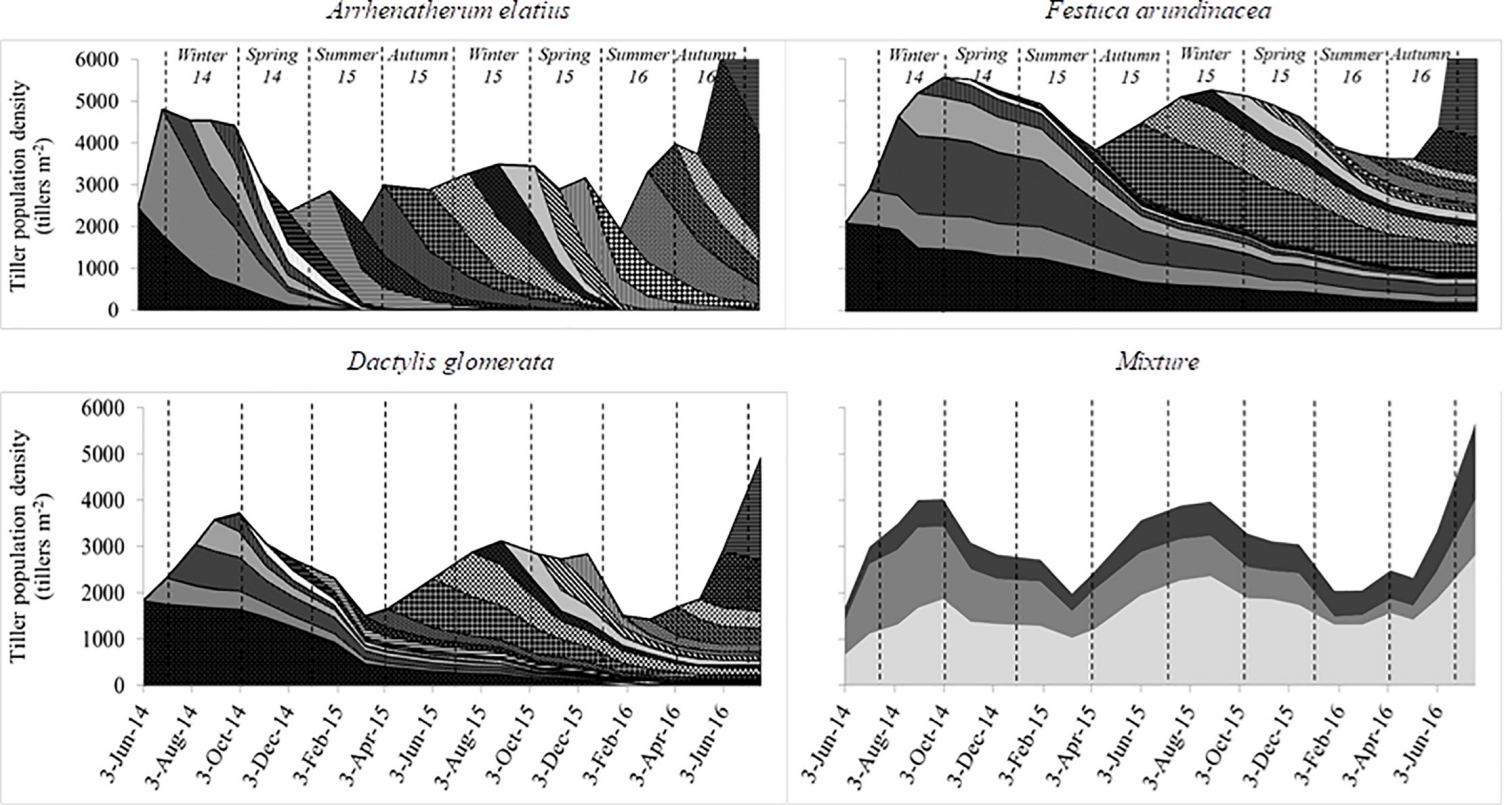
Tillering demography of *Arrhenatherum elatius*, *Festuca arundinacea*, and *Dactylis glomerata* swards sowed as monoculture throughout the experimental period. Different fill patterns represent the tiller density from each of the 22 tiller cohorts. In the mixture, different panel of colours indicate variations in tiller density of *A*. *elatius* (grey), *F*. *arundinacea* (dark grey), and *D*. *glomerata* (light grey) sowed as a mixture over two years.

Diagrams indicating the temporal variations in the tiller density throughout the experimental period were generated to obtain a broader view of the tillering dynamics, indicating the contribution of each tiller cohort (cohorts from *a* to *v*) in monocultures (Fig 1A–1C) and each species in the mixture (Fig 1D). *Arrhenatherum elatius* developed cohorts with many tillers during all seasons, whereas *F*. *arundinacea* and *D*. *glomerata* had numerous cohorts mainly during the autumn and winter. Similar tillering dynamics were observed for the three species growing in monoculture and in the mixture. The proportion of tillers from *D*. *glomerata*, *A*. *elatius*, and *F*. *arundinacea* in the mixture were 52.4%, 24.3%, and 23.0% (*P* < 0.01), respectively, independent of the year (*P* = 0.4766) or season (*P* = 0.9919). Other species, mainly *Holcus lanatus* L., *Paspalum* sp., and *Lolium multiflorum* Lam. contributed up to 0.3% of the total tiller density in the mixture.

### Population changes, tiller mortality, and tiller natality

**Fig 2:**
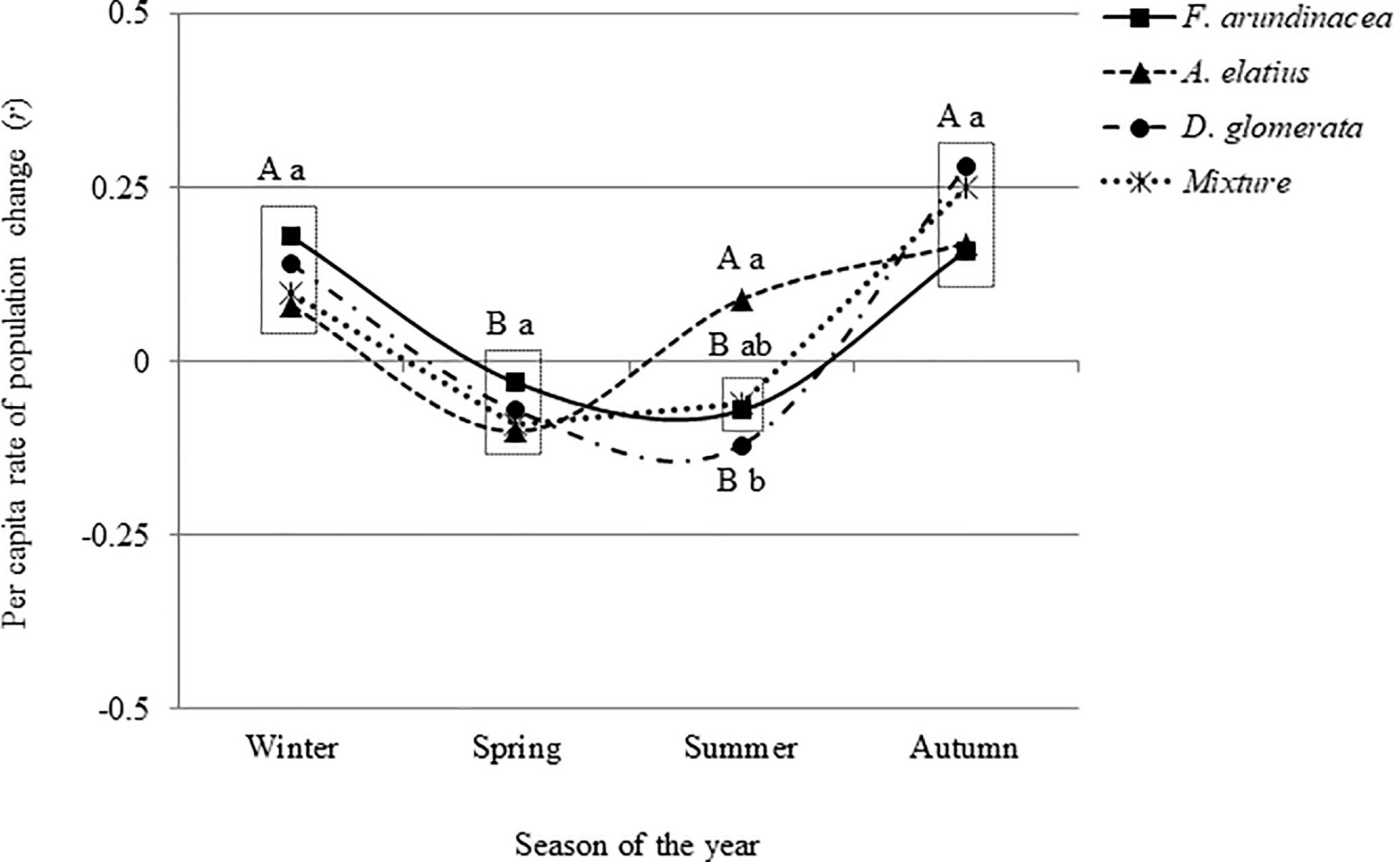
Per capita rate of population change (*r*) in *Arrhenatherum elatius*, *Festuca arundinacea*, and *Dactylis glomerata* swards sowed as monoculture or as a mixture throughout the seasons. Differences among seasons in each treatment are indicated by upper case letters and differences among treatments in the same season are indicated by lower case letters.

There were no year effects for the per capita rate of population change (*r*; *P* = 0.08). All treatments presented similar *r* values throughout winter, spring, and autumn; however, during summer, population of *A*. *elatius* increased more than those of *D*. *glomerata* (*P* < 0.01; Fig 2). Populations of *A*. *elatius* decreased only in spring, whereas the other treatments indicated reduction in their populations during spring and summer. The highest and lowest tiller mortality and tiller natality occurred in *A*. *elatius* and *F*. *arundinacea*, respectively (Table 2). Considering the seasons, *A*. *elatius*, *D*. *glomerata*, and the mixture had the highest tiller mortality during spring and summer, whereas in *F*. *arundinacea* it was constant throughout the year (*P* < 0.01; Table 2). However, the highest tiller natality occurred during the summer and autumn in *A*. *elatius*, autumn and winter in *F*. *arundinacea*, and only during the autumn in *D*. *glomerata* and the mixture (*P* < 0.01; Table 2).

**Table 2:** Tiller mortality and tiller natality in *Arrhenatherum elatius*, *Festuca arundinacea*, and *Dactylis glomerata* swards sowed as monoculture or as a mixture throughout the seasons.

Treatments	Winter	Spring	Summer	Autumn	Mean
	*Tiller mortality (tillers 100 tiller*^*-1*^*30-day-period*^*-1*^*)*				
*Arrhenatherum elatius*	24.2 B a	39.8 A a	40.2 A a	27.6 B a	32.9
*Festuca arundinacea*	5.6 A c	6.1 A c	10.5 A c	9.8 A b	8.0
*Dactylis glomerata*	8.5 B bc	20.0 A b	25.4 A b	13.0 B b	16.7
Mixture	13.6 C b	21.9 AB b	24.2 A b	15.7 BC b	18.9
Mean	13.0	21.9	25.1	16.5	
	*Tiller natality (tillers 100 tiller*^*-1*^*30-day-period*^*-1*^*)*				
*Arrhenatherum elatius*	32.0 B a	30.7 B a	49.2 A a	44.8 AB a	39.2
*Festuca arundinacea*	23.2 A a	3.1 B b	3.5 B c	25.8 A b	13.9
*Dactylis glomerata*	22.4 B a	13.3 B b	13.4 B bc	40.8 A a	22.5
Mixture	23.8 B a	13.1 B b	18.4 B b	40.3 A ab	23.9
Mean	25.4	15.0	21.1	37.9	* *

Means followed by the same uppercase letter in rows and lower case in columns are not significantly different (P > 0.05).

SEM of treatment × season interaction = 1.36 and 3.01 for relative tiller mortality and tiller natality, respectively.

### Number of tillers appearing in each season and their half-life

*Arrhenatherum elatius* produced more tillers in all seasons as compared to the other species (*P* < 0.01; Table 3). *Festuca arundinacea*, *D*. *glomerata*, and the mixture had few tillers appearing during summer and spring; however, the highest number of tillers appeared during winter in *F*. *arundinacea* and during autumn and winter in *D*. *glomerata* and in the mixture (*P* < 0.01). Tillers of *A*. *elatius* and *F*. *arundinacea* had the lowest and highest longevity, respectively, regardless of season (*P* < 0.01; Table 3). The tiller cohorts of *A*. *elatius* that appeared during summer and autumn had longer half-life than those emerged during winter and spring (*P* < 0.01). *Festuca arundinacea* (*P* = 0.39), *D*. *glomerata* (*P* = 0.38), and the mixture (*P* = 0.30) had the same tiller longevity throughout the year.

**Table 3:** Total number of tillers that appeared in each season and half-life of *Arrhenatherum elatius*, *Festuca arundinacea* and *Dactylis glomerata* swards sowed as monoculture or as a mixture throughout the seasons.

Treatments	Winter	Spring	Summer	Autumn	Mean
	*Number of tillers appearing in each season* *(tillers m*^*-2*^*)*				*[155*.*4]*
*Arrhenatherum elatius (276*.*3)*	3930 AB	3177 B	3428 AB	4433 A	3676 a
*Festuca arundinacea (53*.*3)*	2635 A	762 C	350 D	1698 B	1361 c
*Dactylis glomerata (11*.*4)*	1802 A	1414 B	576 C	1868 A	1415 bc
Mixture *(217*.*8)*	2513 A	1590 B	1194 B	2582 A	1970 b
	*Half-life of tiller cohorts appearing in each season (days)*				*[7*.*6]*
*Arrhenatherum elatius (1*.*9)*	36 B	30 B	51 A	50 A	41 c
*Festuca arundinacea (19*.*8)*	213 A	249 A	213 A	248 A	231 a
*Dactylis glomerata (11*.*4)*	120 A	116 A	138 A	109 A	121 b
Mixture *(14*.*5)*	127 A	105 A	105 A	138 A	119 b

Means followed by the same uppercase letter in rows and lower case in columns are not significantly different (P > 0.05).

SEM for season (each pasture alone) and pasture effects are between parenthesis and brackets, respectively.

### Tiller population stability of each species

**Fig 3:**
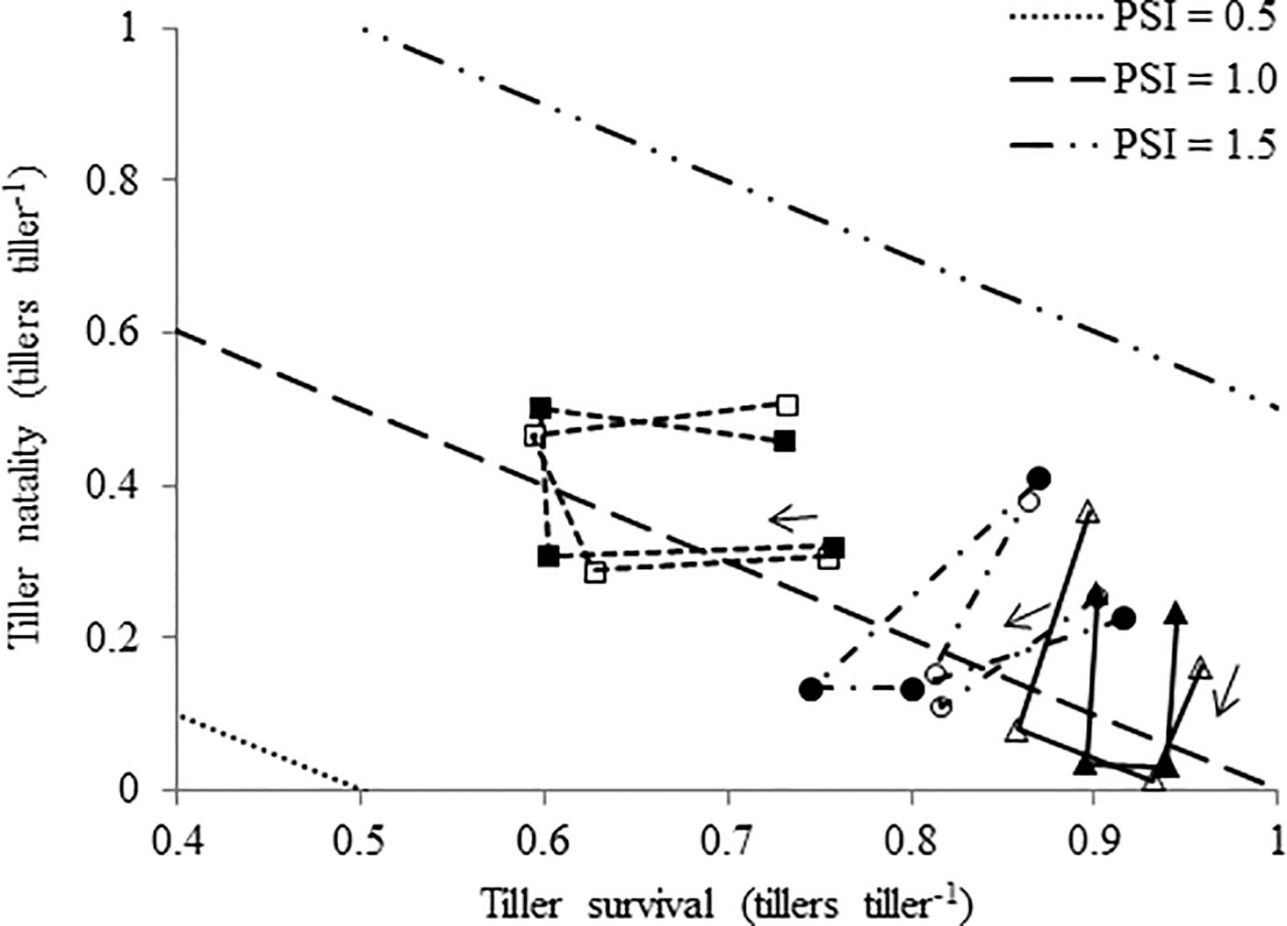
Population stability diagram for *Arrhenatherum elatius* (squares), *Festuca arundinacea* (triangles), and *Dactylis glomerata* (circles) populations sowed as a monoculture (full symbols) or as a mixture (empty symbols) throughout different seasons. The symbols indicate the population stability index (P_1_/P_0_) resulting from the combination of its tiller survival and tiller natality according to the following equation: P_1_/P_0_ = (*b* + *s*)t_1_-t_0_ (adapted from Matthew and Sackville Hamilton, 2011). Arrows indicate the sequence of seasons from winter to autumn.

There were no effects of cultivation method (monoculture or mixture) neither of the interaction cultivation method × season on tiller mortality and tiller natality for the three studied species (P > 0.05; Fig 3). Thus, the cultivation method did not affect the population stability indexes of *A*. *elatius*, *F*. *arundinacea*, and *D*. *glomerata* populations during any season (cultivation method × season interactions: *P* = 0.06, 0.26, and 0.28 for *A*. *elatius*, *F*. *arundinacea* and *D*. *glomerata*, respectively) with mean values of 1.04 (SEM = 0.02, *P* = 0.26), 1.07 (SEM = 0.021, *P* = 0.61), and 1.06 (SEM = 0.016, *P* = 0.51), respectively.

## Discussion

### Relationship between growth and persistence strategies

*Arrhenatherum elatius*, *F*. *arundinacea*, and *D*. *glomerata* monocultures were stable; however, higher mortality and natality of tillers were observed in the most exploitative species (*A*. *elatius*), whereas the conservative species (*F*. *arundinacea*) had lower values. *Dactylis glomerata*, which is a moderately exploitative species, presented intermediate tiller mortality and natality compared to the other two species. These results can be partly explained by the rate of plant tissue renewal, where the most exploitative species have the highest tissue growth and death rates [10]. Consequently, these species produce a greater amount of buds (phyllochron of *A*. *elatius* twice greater than *F*. *arundinacea;* [34]) that are capable of generating new tillers [12]; however, they are less tolerant to stressful conditions [2] and small, young tillers die due to faster development of other tillers [35]. Thus, confirming our first hypothesis, the growth strategy of perennial grasses strongly influences their tillering dynamics and, hence, their persistence strategy when stress and disturbance are minimized. In fact, *A*. *elatius* showed exploitative species characteristics such as high leaf nitrogen content [36], fast tissue turnover, and high specific leaf area (Echeverria, J. R 2017, personal communication) as described by [10, 11]. In contrast, *F*. *arundinacea* presented characteristics of conservative growth strategy species and *D*. *glomerata* had intermediate characteristics [36, 34].

Regardless of the species, tiller densities start to decrease from spring and had a later recovery until winter. Specifically, tiller density recovery starts from summer in *A*. *elatius* and from late autumn in *F*. *arundinacea* and *D*. *glomerata*. [37, 15] also found reductions in tiller density in pastures of *Lolium perenne* and *F*. *arundinacea* (C_3_ species), respectively, during the hottest seasons in subtropical climatic regions. [7] also observed buds dormancy in summer with tillering initiating in autumn for C_3_ grasses in native grasslands. This behaviour observed in *F*. *arundinacea* might be associated with a tillering reduction at higher temperatures and an increase in the tiller number at milder temperatures, especially during shorter photoperiods [38]. According to [39], high temperatures can reduce tillering in *F*. *arundinacea* due to increases in the endogenous auxin concentration at the stem bases. Similarly, [40] found that high temperatures decrease the site usage (number of tillers appearing in relation to the potential buds available for tiller formation; [12]) in C_3_ grasses. Moreover, high tiller density and flowering stimuli during spring (although the employed harvests management strategies minimized inflorescence emissions) may reduce tillering and favour stem elongation and tiller death due to the decapitation of apical meristem [41, 14]. However, [42] indicated that a temporary reduction in tiller density does not necessarily represent a permanent loss of pasture persistence because adjustments between the number and size of tillers can ensure the productive potential of pastures [43]. In fact, heavier tillers with larger leaf area occurred concomitantly with the lowest tiller densities and allowed similar forage yields throughout seasons of the year [44].

Nevertheless, *F*. *arundinacea*, which is a conservative or stress-tolerant species [2, 11], presented extremely low tillering during the hottest seasons of the year (spring and summer), but also had extremely low tiller mortality during these periods. Conversely, *A*. *elatius*, which is an exploitative species, had high tiller turnover also in summer, when the highest temperatures were recorded. According to [45], maintaining high tiller turnover tends to be an inefficient strategy for carbon maintenance in plants; however, that seems typical of exploitative species, which have a grazing resistance strategy based on tolerance mechanisms, and retain young tillers to recover their canopy after defoliation [46]. A consequence of these strategies may be related to the bud bank structure and maintenance, which are tied to the aboveground net primary production and pasture resilience and persistence [7, 8]. Insofar as bud longevity is closely correlated with the aboveground longevity of its parent tiller [6], probably tillering dynamic in *A*. *elatius* is controlled by young tillers (~2–3 months), since they are very short-lived. In contrast, the intense tillering from late autumn and winter in *F*. *arundinacea* were possibly supported by older tillers (more than 6 months), as few tillers were recruited in summer and late spring. Moreover, as *A*. *elatius* used their bud bank more frequently to recruit new tillers that contribute to support its biomass production throughout the year, their bud bank may become too small to recover tiller population after adverse conditions and its long-term persistence could be harmed if stresses and/or disturbances are intensified hereafter. Lastly, it is expected that the species studied here present different vegetative life-histories and, consequently, different long-term responses to stresses and disturbances [13, 6].

As discussed above, the contrasting tiller turnover among the studied species support the idea that exploitative species present persistence strategies based on the appearance of new tillers, whereas conservative species persist by prioritising the survival of existing tillers and are in agreement with the growth strategies proposed by the ‘*r-K* continuum’ [1] and the ‘three primary strategies’ models [47, 2]. This result might be one of the reasons why [48] found a relationship between dominant species and their respective stress tolerance and competitive abilities in which fertile environments favoured *A*. *elatius* and poorer environments were dominated by *Bromus erectus* (a more conservative species). Likewise, [26] found that in fertile environments the conservative and exploitative species had the same proportion, whereas in non-fertilised pastures 80 ± 8% of the total biomass was composed of conservative species. This is possible because nutrient availability affects plant growth more than plant survival [49], and the high tiller mortality in exploitative species cannot be compensated by high tiller natality once the buds start competing with the apical meristem when there is resource limitation [50].

Tiller half-life is extremely variable among species and tiller cohorts [16], ranging from 36 to 143 days for *L*. *perenne* [33] and from 321 to 902 days for *Paspalum notatum* [51]. Although growth strategies have not been discussed in these studies, it seems that *L*. *perenne* plants have a grazing resistance strategy based on tolerance (more associated with exploitative species), whereas the strategy of *P*. *notatum* is more related to avoidance mechanisms (typical of conservative species) [45]. [48] also observed lower tiller survival in *A*. *elatius* than in *Brachypodium rupestre* and *B*. *erectus* (more conservative species). Likewise, in the present study, the half-life of tillers increased from the most exploitative to the more conservative species. In this sense, [17] suggested that species with very long-lived tillers might have lower seasonal variations in tiller density. However, our results suggest that in environments with low resource limitations exploitative species can compensate for their low tiller longevity with high tiller natality and also create stable pastures throughout the year, although through different mechanisms.

### Tillering dynamics of populations sowed as monocultures or as a mixture

Although tiller density in the mixture was slightly higher than of *D*. *glomerata*, its tillering dynamics were similar. This result was because *D*. *glomerata* represented 52.4% of the total tillers present in this treatment and tiller mortality and natality were intermediate to *A*. *elatius* and *F*. *arundinacea*, which equally contributed to the tiller density of the mixture. Tiller mortality and natality are controlled by several factors, some of which are intrinsic to the species and their development whereas others result from the environment in which these tillers develop, such as nutrient availability, light quality and intensity [52, 14]. Several studies have found different influences of intra- and inter-specific competition on tiller dynamics [53, 54]. However, these results were mainly a response to the different sward structures created by the presence of species with different sizes, architectures, and/or growing period throughout the year. In this regard, our results suggest that when perennial winter grass species grow in conditions of low levels of stress and disturbance, their tillering dynamics are mainly determined by genet intrinsic factors. Therefore, supporting our second hypothesis, the populations of *A*. *elatius*, *F*. *arundinacea*, and *D*. *glomerata* studied here showed the same tillering dynamics whether in monoculture or in a mixture of them.

## Conclusion and implications

The results from our study support the hypothesis that perennial grass species with exploitative and conservative growth strategies also have different strategies for population persistence when managed to minimize stress and disturbance. The most exploitative species (*A*. *elatius*) have high tiller mortality during all seasons of the year and are extremely dependent on numerous successive tiller cohorts to persist while more conservative species (*F*. *arundinacea*) have longer-lived tillers and recovery its tiller density at specific time of the year. Therefore, it is necessary to ensure a constant and adequate input of resources for tillering to make exploitative species persistent. However, although conservative species are less affected by stresses, adequate resource supply at the season when their recover its tiller density could help for their long-term persistence, once this species need that many tillers emerge at that time. In addition, managements that minimize stress and disturbance allow similar tillering dynamics between populations growing in monoculture or mixture. This indicates the possibility of using multispecies pastures in intensive animal production systems without any damage to the persistence of sowed species with the benefits of a more diverse grassland.

## Supporting information

S1 Fig Climate variables throughout the experimental period and mean values for the last 85 years in Lages, Santa Catarina, Brazil. Source: Centro de Previsão de Tempo e Estudos Climáticos do Instituto Nacional de Pesquisas Espaciais–CPTEC/INPE.
(DOCX)

S1 Table Fertilisation activities performed throughout the experimental period. (XLSX)

S1 Data Data collected in the field and used to proceed all the analysis and create the figures. (XLSX)
